# Spraying Foliar Fertilizer Affect the Physiological Function of Leaf and Improve the Quality of ‘*Snick*’ Apple

**DOI:** 10.3390/plants14182926

**Published:** 2025-09-20

**Authors:** Hong-Fu Xu, Shi-Mei Li, Wei-Feng Ma, Shi-Xiong Lu, Zhi-Yuan Bian, Guo-Ping Liang, Juan Mao

**Affiliations:** The College of Horticulture, Gansu Agricultural University, Lanzhou 730070, China; 18893151879@163.com (H.-F.X.); 15214038359@163.com (S.-M.L.); 18409490212@163.com (W.-F.M.); 18893912407@163.com (S.-X.L.); lzubzy@163.com (Z.-Y.B.); lianggp@gsau.edu.cn (G.-P.L.)

**Keywords:** ‘Snick’ apple, foliar fertilizer, leaf physiological, fruit quality, aroma components

## Abstract

Foliar fertilizers are efficient in enhancing nutrient utilization. This experiment aims to improve leaf physiological functions, enhance fruit quality, increase yield, and boost orchard productivity through the screening of foliar fertilizers suitable for apple trees. The 6-year-old apple trees of the ‘Snick’ were used as experiment material. The results of measurements amino acids, calcium, boron, and potassium indicate that different foliar fertilizers can improve fruit quality and aroma by enhancing leaf physiological functions. In apple fruit, amino acid foliar fertilizer increased the tartaric acid content by 44.26%. Calcium foliar fertilizer resulted in a 32.39% increase in vitamin C, a 19.71% increase in sucrose compared to the control, with a total aroma substance increase of 13.41%. Boron foliar fertilizer elevated flavonoid content in the peel to 3.67 mg·g^−1^, a 70.69% increase over the CK. Potassium foliar fertilizer significantly improved fruit appearance, phenolic substances in the peel, soluble protein content by 25.39%, and glucose content by 55.91%. Therefore, mineral source fulvic acid potassium foliar fertilizer was demonstrated the best overall effect, effectively enhancing fruit quality and flavor. These results provide a theoretical basis and scientific reference for improving apple quality.

## 1. Introduction

Apple (*Malus domestica* Mill.) belongs to the Rosaceae apple genus [1], the world planting area is about 4 to 5 million hectares, the output is about 79 million tons [2]. The Loess Plateau (Latitude ranges from 33°41′ N to 41°16′ N, and longitude spans from 100°52′ E to 114°33′ E) has become a central apple-producing area globally and serves as a key region for the high-quality cultivation of apples in the world [3], it accounts for over 25% of China’s apple production [4]. It is a densely cultivated fruit [5], mainly freshly eaten. Its market value is affected by factors such as color, shape, hardness [6]. At the same time, it has become one of the most important fruit crops in the economy and nutritional value due to its excellent taste and rich functional components [7].

Foliar fertilizers, as environmentally friendly fertilizers that are not directly applied to the soil, according to the fertilizer brands primarily contain growth regulators, small-molecule organic acids, and other components [8]. Compared to root fertilization, foliar fertilizers are applied by spraying plant leaves, enabling faster nutrient absorption [9]. Under optimal conditions, they can effectively improve the fruit quality and yield of various horticultural crops [10], but may cause damage to plant leaves. It is recommended to use low-concentration fertilizers [11].

Photosynthesis is the most important chemical reaction in plants [12] and is influenced by external environmental conditions such as light, temperature, humidity, carbon dioxide concentration [13], mineral elements [14], hormones [15], and leaf development [16]. Research shows that increasing the rate of net photosynthesis is crucial to crop yield and quality, so optimizing the photosynthetic mechanism is a key strategy for improving fruit quality with foliar fertilizers [17].

Fruit quality encompasses external attributes such as weight, vertical and horizontal diameters, peel colour, and internal attributes like soluble solids content, vitamin C, soluble sugars, and titratable acidity. Various factors, including nutrient elements such as boron, calcium, potassium, and amino acids, influence this quality. Amino acid foliar fertilisers, high in amino acids and trace elements, can rapidly penetrate plant tissues. Consequently, they promote growth and enhance the content of vitamin C, total phenolic compounds, total flavonoids, total sugars, and soluble solids in fruits [18]. Calcium, an activator of many essential enzymes in plants, enhances enzyme activity and promotes various chemical reactions, thereby regulating plant growth and development. Calcium sprays significantly affected the single fruit weight, fruit flesh firmness, peel color, soluble solids content, soluble sugar, titratable acid, soluble protein, and vitamin C content of the fruit [19]. Plants supplied with sufficient boron can increase the supply of organic matter in various organs, enabling normal crop growth and increasing seed and fruit setting rates. The results showed that spraying boron increased fruit size (diameter, length, weight) and fruit quality (colour and firmness) [20]. Moreover, it promotes the accumulation of soluble solids, protein, vitamin C, total phenol, flavonoids, esters, and other characteristic compounds. As free ions, potassium plays a crucial role in plants by enhancing carbohydrate and nitrogen metabolism [21]. Potassium fertilizer has increased fruit weight, soluble solids, total sugar content, sugar-acid ratio, vitamin C, and phenolic substances. Therefore, it is evident that different nutrient elements affect fruit quality differently [22].

The quality of apple directly impacts their commercial value, and market competitiveness. To maximize economic benefits, improving apple quality such as size, shape, colour, absence of bruising and other is essential. In this study, ‘Snick’ apples were used as the experiment materials, based on the type and cost of the foliar fertilizer, different concentrations and application timings were used for leaf spraying, followed by analyzing the fruits for external appearance and nutritional quality. Additionally, the aromatic compounds in the fruits were examined. This study aims to select foliar fertilizers suitable for ‘Snick’ apples that can improve fruit quality, and establish a foundation for further research into the efficacy and mechanisms of foliar fertilizer applications. Moreover, we will also focus on the development and investigation of novel foliar fertilizers, including nano-formulated, specialty, and chelated foliar fertilizers.

## 2. Results

### 2.1. Effect of Different Foliar Fertilizers on Physiological Characteristics of Shoots and Leaves of ‘Snick’ Apple

**Figure 1:**
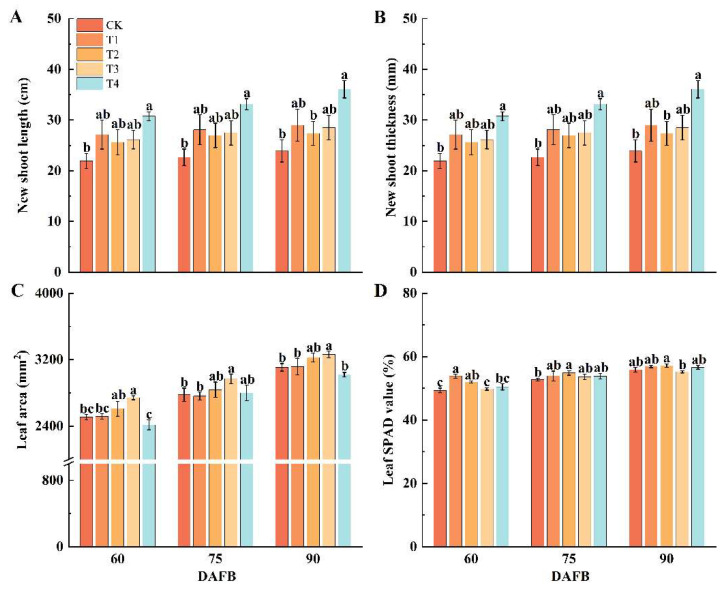
Effect of different foliar fertilizers on physiological characteristics of shoots and leaves of ‘Snick’ apple. DAFB: Days after full bloom. (**A**): New shoot length. (**B**): New shoot thickness. (**C**): Leaf area. (**D**): Leaf SPAD value. CK: Clear water control. T1: Amino acid foliar fertilizer. T2: Calcium foliar fertilizer. T3: Boron foliar fertilizer. T4: Potassium foliar fertilizer. Error bars represent the mean ± SE from three biological repeats. Different lowercase letters denote significant differences, whereas the same lowercase letters indicate no statistical difference (*p* < 0.05).

The results showed that as the number of days after full bloom (DAFB) increased, both the length and diameter of new shoots exhibited a gradually increasing trend. By 90 DAFB, under the mineral source potassium fulvic acid (T4), the new shoot length reached 36.06 cm, representing a significant increase of 50.81% compared with the CK treatment; the new shoot diameter was 6.71 mm, showing a significant increase of 11.22% relative to CK. From 60 to 90 DAFB, leaf area displayed an upward trend, with the maximum leaf area observed under the sugar alcohol boron (T3) at 90 DAFB (3263.18 mm^2^), which was 5.03% higher than CK, but the leaf area under the T4 was smaller than that of the CK between 60 and 90 DAFB. By 90 DAFB, the leaf area in T4 had decreased by 2.81% compared to CK, suggesting that the T4 may partially inhibit leaf area expansion. This possibly results from a balance between shoot elongation and leaf expansion. This was followed by the effect of fluid calcium (T2), with a 3.83% increase compared to CK. At 60 DAFB, the SPAD value of leaves under the Ye Xiumei (T1) was 53.84%, 9.18% higher than CK. Notably, the T2 achieved the highest SPAD values at both 75 DAFB and 90 DAFB, with increases of 4.03% and 2.46% over CK, respectively. These results indicate that the T2 treatment may enhance the photosynthetic potential of the leaves (Figure 1D).

### 2.2. Effect of Different Foliar Fertilizers on Photosynthesis in ‘Snick’ Apple Leaves

**Figure 2:**
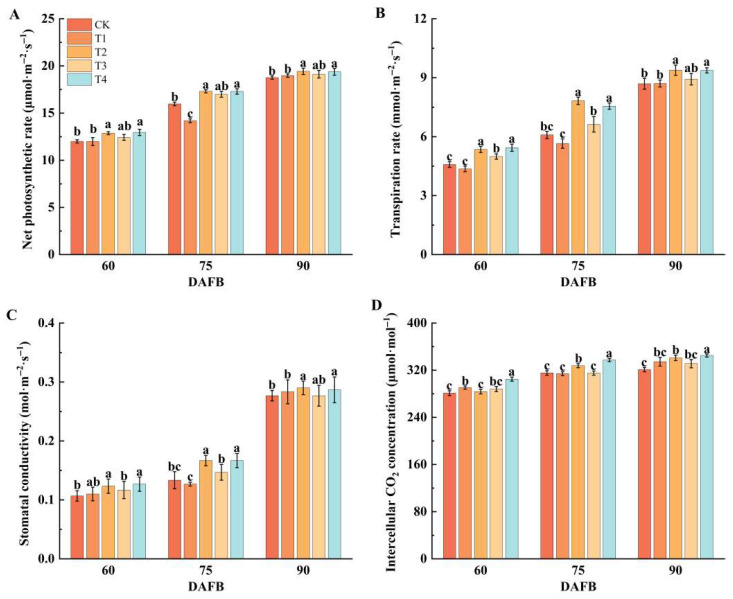
The effect of different foliar fertilizers on photosynthesis in ‘Snick’ apple leaves. DAFB: Days after full bloom. (**A**): Net photosynthetic rate. (**B**): Transpiration rate. (**C**): Stomatal conductivity. (**D**): Intercellular CO_2_ concentration. CK: Clear water control. T1: Amino acid foliar fertilizer. T2: Calcium foliar fertilizer. T3: Boron foliar fertilizer. T4: Potassium foliar fertilizer. Error bars represent the mean ± SE from three biological repeats. Different lowercase letters denote significant differences, whereas the same lowercase letters indicate no statistical difference (*p* < 0.05).

The results showed that from 60 to 90 DAFB, the net photosynthetic rate, transpiration rate, stomatal conductance, and intercellular CO_2_ concentration of leaves under all treatments exhibited an upward trend (Figure 2). According to the Figure 2A, at 90 DAFB, the net photosynthetic rate of the T2 reached 19.42 μmol·m^−2^·s^−1^, representing a 3.59% increase compared with the CK. The results indicate that the T2 treatment significantly enhanced the net photosynthetic rate. The transpiration rate reached 9.38 mmol·m^−2^·s^−1^, a 7.97% increase over CK, and the stomatal conductance was 0.29 mol·m^−2^·s^−1^, an increase of 4.81% relative to CK, calculation of intrinsic water-use efficiency (PN/gs) revealed a significant decrease in intrinsic water-use efficiency as the days after flowering increased. However, the T4 treatment effectively delayed this decline, exhibiting a reduction rate that was 22.41% slower compared to the CK. The intercellular CO_2_ concentration was 344.96 μmol·mol^−1^, an increase of 7.51% compared with CK.

### 2.3. Effect of Different Foliar Fertilizers on Fruit Appearance Quality at Harvest Time

**Figure 3:**
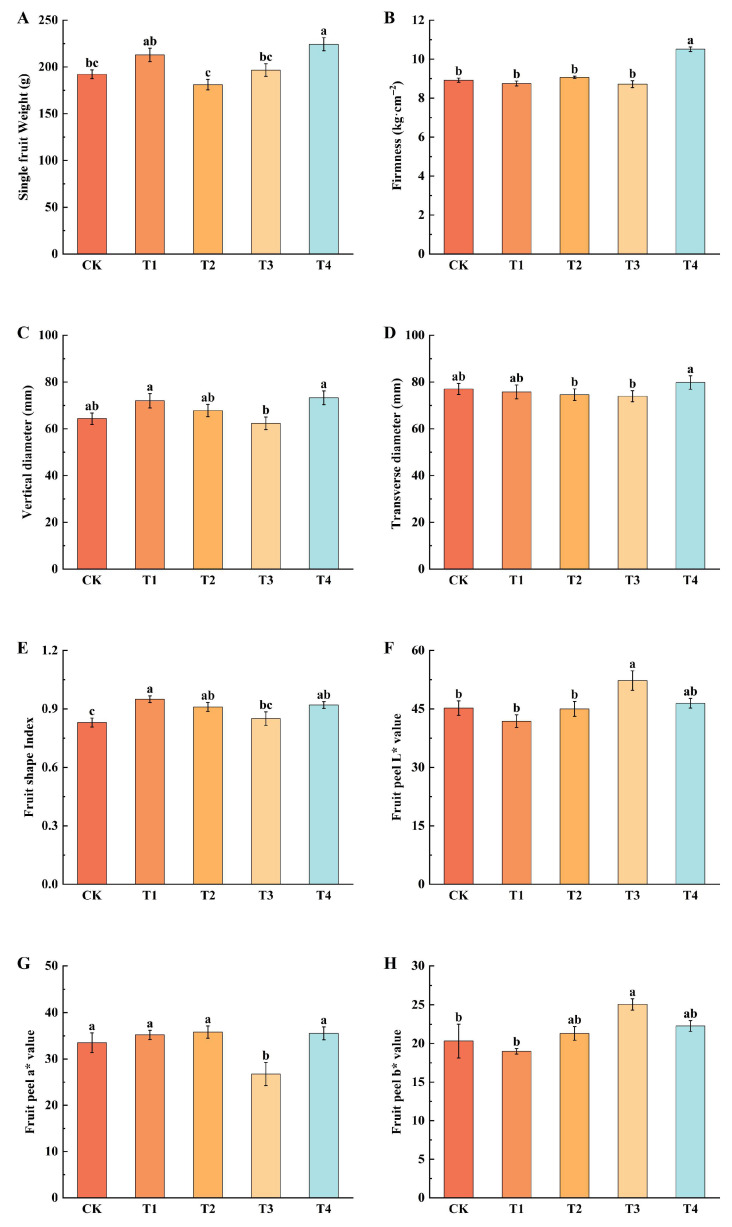
Effect of different foliar fertilizers on fruit appearance quality. (**A**): Single fruit Weight. (**B**): Firmness. (**C**): Vertical diameter. (**D**): Transverse diameter. (**E**): Fruit shape Index. (**F**): Fruit peel L* value. (**G**): Fruit peel a* value. (**H**): Fruit peel b* value. CK: Clear water control. T1: Amino acid foliar fertilizer. T2: Calcium foliar fertilizer. T3: Boron foliar fertilizer. T4: Potassium foliar fertilizer. Error bars represent the mean ± SE from three biological repeats. Different lowercase letters denote significant differences, whereas the same lowercase letters indicate no statistical difference (*p* < 0.05).

The results showed that the single fruit weight (224.27 g), hardness (10.51 kg·cm^−2^), longitudinal diameter (73.26 mm), and transverse diameter (79.80 mm) of T4 treatment were significantly higher than those of CK (Figure 3), increasing by 16.69%, 17.83%, 13.89%, and 3.58%, respectively. The fruit shape index of T1 and T4 was significantly higher than that of other treatments. The fruit shape indexes of CK and T3 ranged between 0.8 and 0.9, indicating nearly round fruits, while those of T1, T2, and T4 ranged between 0.9 and 1.0, indicating more elongated fruits. The highest L* value of the fruit peel was observed in T3, followed by T4, the L* value of CK was higher than those of T2 and T1. The highest a* value component in T4 and the lowest in T3. The b* values for T2, T3, and T4 were higher than CK, whereas the b* value for T1 treatment was lower than CK.

### 2.4. Effect of Different Foliar Fertilizers on Fruit Internal Quality

#### 2.4.1. Effect of Different Foliar Fertilisers on the Soluble Solids, Soluble Sugar, Titratable Acid and Sugar-Acid Ratios of Fruits

**Figure 4:**
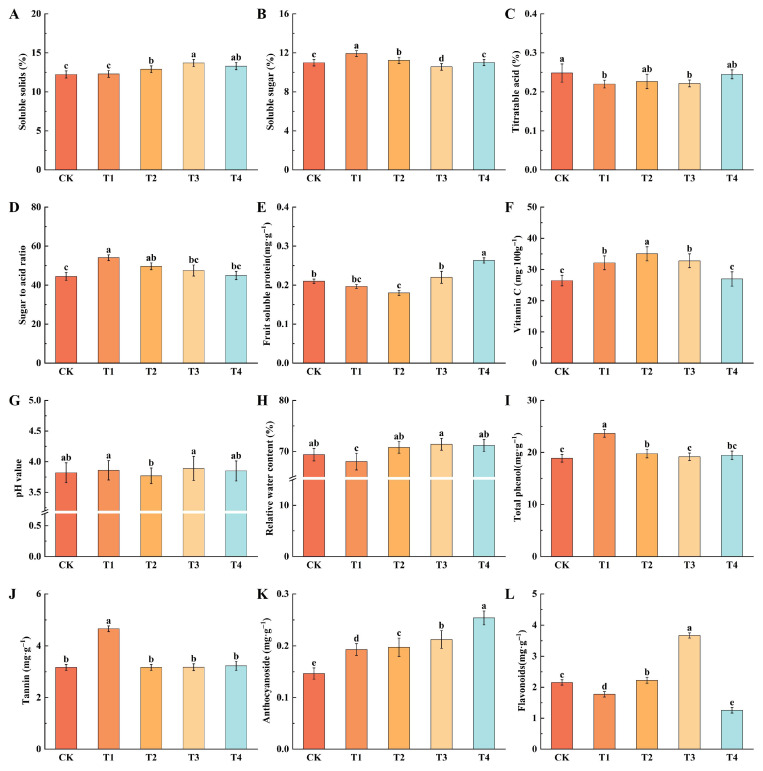
Effect of different foliar fertilizers on fruit internal quality. (**A**): Soluble solids content. (**B**): Soluble sugar content. (**C**): Titratable acid content. (**D**): Ratio of sugar to acid. (**E**): Fruit soluble protein content. (**F**): Vitamin C content. (**G**): pH value. (**H**): Relative water content. (**I**): Total phenolic content. (**J**): Tannin content. (**K**): Anthocyanin content. (**L**): Flavonoid content. CK: Clear water control. T1: Amino acid foliar fertilizer. T2: Calcium foliar fertilizer. T3: Boron foliar fertilizer. T4: Potassium foliar fertilizer. Error bars represent the mean ± SE from three biological repeats. Different lowercase letters denote significant differences, whereas the same lowercase letters indicate no statistical difference (*p* < 0.05).

The results indicated that applying foliar fertilizer increased the soluble solids content, with the T3 showing the most significant effect at 13.71%, which was 12.01% higher than the CK (Figure 4A). Under the T1, the apple fruits had the highest soluble sugar content and the lowest titratable acid content, suggesting that T1 effectively promotes the accumulation of soluble sugars while reducing titratable acid levels (Figure 4B,C). Further analysis of the sugar-acid ratio revealed that the T1 resulted in a sugar-acid ratio of 54.07, a 21.56% increase compared to the CK (Figure 4D). These results indicated that amino acid foliar fertilizer could promote the accumulation of sugar and the increase of sugar acid ratio in fruit, boron foliar fertilizer could increase the accumulation of soluble solids, and they all reduced the accumulation of titratable acid.

#### 2.4.2. Effect of Different Foliar Fertilizers on Soluble Protein, Vitamin C Content, pH Value and Relative Water Content of Fruits

Further investigation into the impact of various foliar fertilizers on soluble protein content revealed that T3 and T4 had higher levels than the CK, whereas T1 and T2 led to reduced soluble protein concentrations (Figure 4E). Specifically, the soluble protein content under T4 was 0.26%, marking a significant 25.39% increase relative to CK. Additionally, application of foliar fertilizers enhanced vitamin C content in the fruit, the most effective treatment was T2, followed by T3, T1, and T4, with all of these being more effective than CK, representing increases of 32.39%, 23.91%, 21.42%, and 1.91% over CK, respectively(Figure 4F). Regarding pH values and relative water content, T3 exhibited the highest values at 3.89 and 71.42%, respectively, though these differences were not statistically significant compared to the CK (Figure 4G,H).

#### 2.4.3. Effect of Different Foliar Fertilizers on the Concentrations of Total Phenolic Compounds, Tannins, Anthocyanins, and Flavonoids

The changes of total phenol and tannin content under different foliar fertilizer treatments were analyzed. The T1 resulted in the highest total phenol content (23.66 mg·g^−1^) and tannin content (4.66 mg·g^−1^), which were 25.38% and 47.01% higher than the CK, respectively (Figure 4I,J). In contrast, no significant differences in total phenol and tannin contents were observed under other treatments compared to the CK. Following the application of foliar fertilizers, all treatments exhibited increased anthocyanin content relative to the CK, with the T4 yielding the highest anthocyanin content at 0.25 mg·g^−1^, representing a 73.09% increase over the CK (Figure 4K). Regarding flavonoid content, different fertilizers had varying effects: the T3 resulted in the highest content (3.67 mg·g^−1^), marking a 70.69% increase from the CK, the T4 produced the lowest content (1.26 mg·g^−1^) (Figure 4L).

### 2.5. Effect of Different Foliar Fertilizers on the Sugar and Acid Components of Fruits

**Figure 5:**
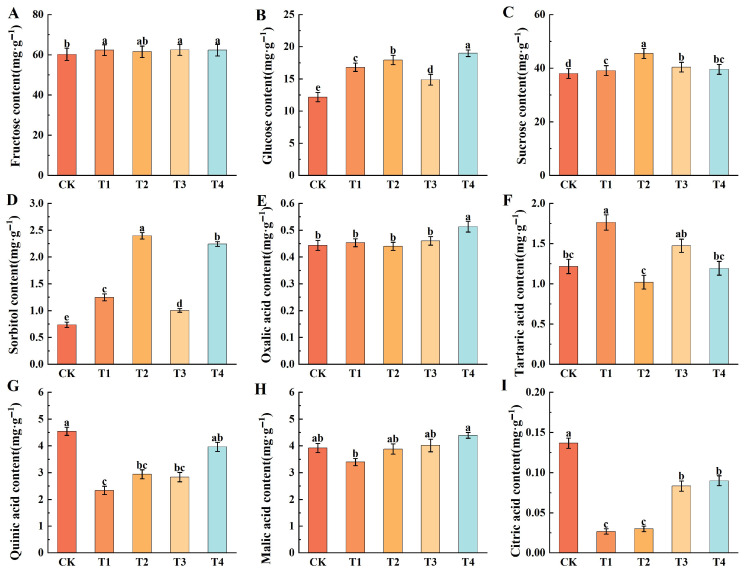
Effect of different foliar fertilizers on the sugar and acid components of fruits. (**A**): Fructose content. (**B**): Glucose content. (**C**): Sucrose content. (**D**): Sorbitol content. (**E**): Oxalic acid content. (**F**): Tartaric acid content. (**G**): Quinic acid content. (**H**): Malic acid content. (**I**): Citric acid content. CK: Clear water control. T1: Amino acid foliar fertilizer. T2: Calcium foliar fertilizer. T3: Boron foliar fertilizer. T4: Potassium foliar fertilizer. Error bars represent the mean ± SE from three biological repeats. Different lowercase letters denote significant differences, whereas the same lowercase letters indicate no statistical difference (*p* < 0.05).

Analysis of the sugar components revealed that fructose content was the highest, followed by sucrose and glucose, with sorbitol content being the lowest. The effects of different foliar fertilizers on these sugar components indicated no significant differences in their impact on fructose levels. Among the treatments, T4 showed the highest fructose content at 62.29 mg·g^−1^, a 3.42% increase over the CK. Foliar fertilizers increased glucose, sucrose, and sorbitol contents in the fruit. Specifically, T4 resulted in the highest glucose content at 18.99 mg·g^−1^, representing a 55.91% increase compared to the control. Additionally, T2 yielded the highest sucrose (45.50 mg·g^−1^) and sorbitol (2.40 mg·g^−1^), which were 19.71% and 324.32% higher than the CK, respectively (Figure 5A–D).

Further analysis of the impact of various foliar fertilizers on fruit acid composition revealed that oxalic acid (0.51 mg·g^−1^) levels were highest under T4, an increase of 15.91% compared to the CK. Malic acid is a widely prevalent organic acid, and was consistently identified as one of the primary organic acids across all analyzed samples in this study. Under T4 treatment, its content reached 4.39 mg·g^−1^, representing a 11.99% increase over the CK. Conversely, tartaric acid content peaked under T1 at 1.76 mg·g^−1^, marking a 44.26% elevation over CK. In contrast, quinic acid and citric acid exhibited declining trends relative to CK, with their contents under T1 being the lowest, at 2.34 mg·g^−1^ for quinic acid and 0.03 mg·g^−1^ for citric acid (Figure 5E–I).

### 2.6. Effect of Different Foliar Fertilizers on Aroma Composition of Apple Fruits

#### 2.6.1. Effects of Different Foliar Fertilizers on the Contents of Aldehydes, Esters, Alcohols and Other Substances in Aroma Components

**Figure 6:**
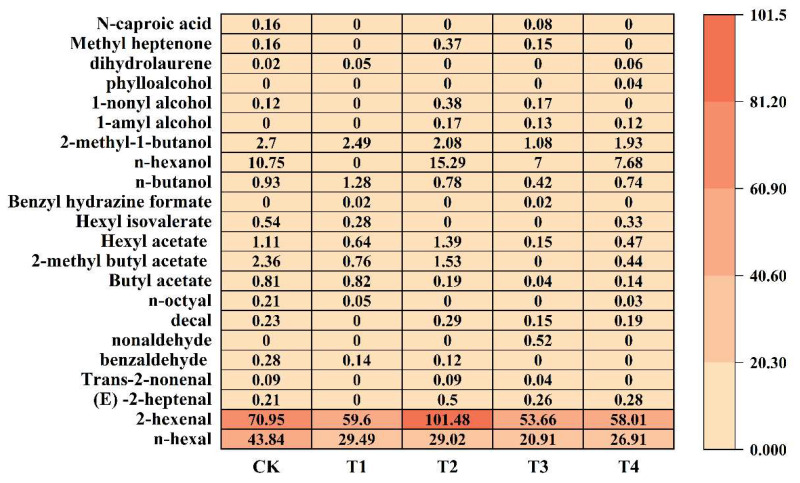
The content of aroma substances in fruits treated with different foliar fertilizers, the abscissa is the treatment, and the ordinate is the name of the aroma substance. The depth of the color represents the level of aroma content. The number in the figure represents the volatile aroma content, and the unit is μg·kg^−1^. CK: Clear water control. T1: Amino acid foliar fertilizer. T2: Calcium foliar fertilizer. T3: Boron foliar fertilizer. T4: Potassium foliar fertilizer.

Through the analysis of aroma substances in fruits, 25 primary aroma compounds were identified and categorized into six groups: eight aldehydes, six esters, six alcohols, one aromatic hydrocarbon, one ketone, and one acid. These aroma substances were distributed across different treatments: 23.89%, 16.87%, 27.11%, 14.95%, and 17.18% of the total aroma content, respectively. Specifically, the proportions of aldehydes, esters, alcohols, aromatic hydrocarbons, ketones, and acids within each treatment relative to the total aroma content were 87.76%, 2.12%, 9.92%, 0.03%, 0.12%, and 0.05%, respectively (Figure 6).

Analysis of various aromatic compounds in apple fruit revealed that hexanal and 2-hexanal were the most abundant aldehydes. Among these, CK exhibited the highest hexanal content, whereas hexanal levels in other treatment groups were significantly lower, indicating that foliar fertilizer application reduces hexanal production in apple fruit. The 2-hexenal content in T2 was significantly higher than in CK, reaching 101.48 μg·kg^−1^, a value 43.03% greater than the control group. Ethyl butyrate, ethyl 2-methylbutyrate, and ethyl hexanoate were the most abundant ester compounds in apple fruit. Ethyl 2-methylbutyrate, the most abundant ester, was not detected in T3. The ethyl butyrate content in T1 was not significantly different from CK, whereas it was lower in other treatment groups. In T2, ethyl hexanoate content was significantly higher than in CK but significantly lower than CK in other treatment groups. n-Hexanol was the most abundant alcohol in apple fruit, though it was not detected in the T1 treatment group. The n-hexanol content in T2 was significantly increased by 42.23% compared to CK, whereas it was significantly lower than CK in other treatment groups. In addition to aldehydes, esters, alcohols, apple fruit also contains olefins, ketones, and acids. Following the application of foliar fertilizer. Dihydromyrcene content increased in T1 and T4, while methyl heptanone content rose significantly in the T2, reaching 0.37 μg·kg^−1^, a 131.25% increase compared to the CK. Hexanoic acid was present only in the CK and T3, with the T3 showing a lower content than the CK.

#### 2.6.2. Changes of Total Aroma Substance Content and Characteristic Aroma Under Different Nutrient Foliar Fertilizer Treatments

**Figure 7:**
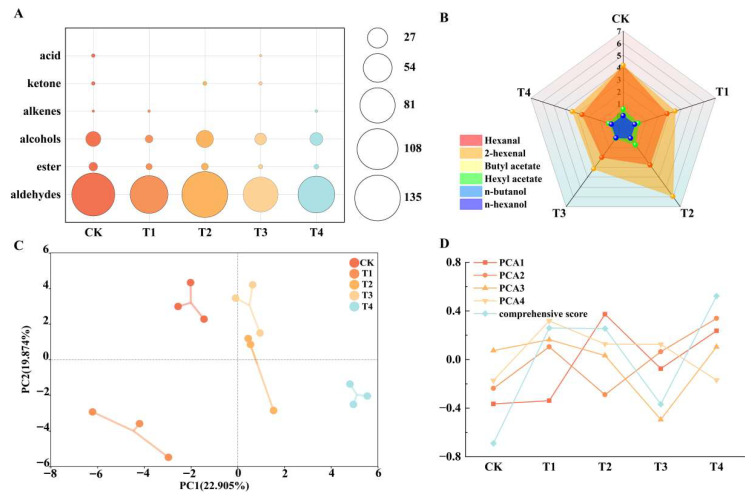
Analysis of aroma content and principal component analysis of determination indexes. (**A**): The total content of aldehydes, esters, alcohols, olefins, ketones and acids aroma substances under different nutrient elements foliar fertilizer treatment, the size of the circle represents the content of aroma substances. (**B**): The content of characteristic aroma under different nutrient element foliar fertilizer treatment. (**C**): Centroid PCA analysis of determination index. (**D**): The scores and comprehensive scores of each treatment on different main analyses. CK: Clear water control. T1: Amino acid foliar fertilizer. T2: Calcium foliar fertilizer. T3: Boron foliar fertilizer. T4: Potassium foliar fertilizer.

The primary aroma substances in apple fruit were aldehydes, which accounted for the highest proportion, followed by alcohols and then aromatic hydrocarbons (Figure 7A). Among these, T2 significantly increased the total content of apple aroma substances by 13.41% compared to the CK. Specifically, under T2, the contents of aldehydes, alcohols, and ketones increased significantly, while other aroma substances decreased compared to the CK.

The aroma components with a value greater than 1 are considered characteristic of the fruit and play a significant role in its overall aroma profile. n-hexanal and 2-hexenal are key characteristic aroma components within the aldehyde category, exhibiting aroma values exceeding one across all treatments, including the CK (Figure 7B). Aldehydes and alcohols were the predominant volatile aroma substances in apples subjected to various foliar fertilizer treatments. Esters typically imparted sweet aromas, while aldehydes and alcohols contributed to green aromas. Notably, ‘Snick’ apple fruits displayed the highest aldehyde content, it is speculated that the variety is mainly fresh green aroma.

### 2.7. Principal Component Analysis and Correlation Analysis

#### 2.7.1. Principal Component Analysis

To comprehensively assess the impact of various foliar nutrient fertilizers on ‘Snick’ apples, Principal Component Analysis was employed on the 42 measured indicators (Figure 7C). This analysis yielded four principal components (PC1–PC4), which collectively explained 100% of the variance, thereby validating their use as a condensed representation of the original dataset for evaluating foliar fertilizer effects. Each principal component’s eigenvalue served as the weight for calculating the principal component score, facilitating the computation of a comprehensive correlation score. This score enabled ranking of treatments based on their overall enhancement of apple quality and aromatic profiles, with higher scores indicating superior treatment efficacy. Following the application of principal component analysis and subsequent calculations, the effectiveness ranking of various foliar fertilisers on snick apple quality was determined as follows: among all treatments, T4 exhibited the highest improvement effect, followed by T1, then T2 and T3, though all treatments significantly outperformed the CK (Figure 7D).

#### 2.7.2. Analysis of Relationship

Analysis of leaf physiological parameters revealed significant positive correlations: between new shoot length and new shoot thickness (r = 0.92, * *p* < 0.05), between net photosynthetic rate and transpiration rate (r = 0.96, * *p* < 0.05). Further analysis of fruit quality showed highly significant positive correlations (between peel L* value and peel b* value, r = 0.98, ** *p* < 0.01), a highly significant negative correlation (between pH value and aroma compounds, r = −0.97, ** *p* < 0.01), and a highly significant positive correlation (between total phenols and tannins, r = 0.99, ** *p* < 0.01).

**Figure 8:**
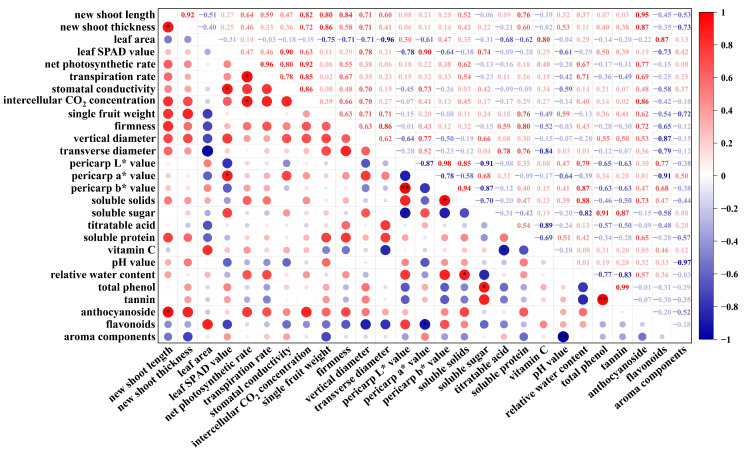
Correlation heat map. The intra-group correlation between main leaves and quality. Red represents a positive correlation, blue represents a negative correlation, and the depth of the color represents the size of the correlation coefficient. The size of the circle represents the strength of the correlation. Pearson correlation coefficient (r): measures the linear correlation between two continuous variables, from negative to positive. (−1 < r < 1) *p*: To test whether the r value is significant; * *p* < 0.05, significant correlation, ** *p* < 0.01, extremely significant correlation.

Investigations into correlations between leaf and fruit physiological indicators (Figure 8) further revealed: a significant positive correlation between new shoot length and anthocyanins (r = 0.95, * *p* < 0.05); a significant negative correlation between leaf area and transverse diameter (r = 0.96, * *p* < 0.05). These leaf-fruit correlations indicate that foliar fertilizer application influenced leaf physiological functions, which in turn affected fruit quality.

## 3. Discussion

Foliar fertilization primarily stimulates plant growth and boosts resilience against environmental stresses, ultimately improving crop yield and quality [23]. Key determinants of fruit sensory quality include the concentrations of soluble solids, titratable acidity, soluble sugars, and vitamin C [24]. Additionally, flavonoids, phenolic compounds, and soluble proteins are pivotal indicators associated with peel coloration, antioxidant capacity, and physiological metabolic activity [25].

Leaves are one of the nutritional organs of vascular plants. Their morphological structure is highly susceptible to changes in ecological conditions, enabling adaptation to their environment [26]. Their primary functions include carrying out photosynthesis to synthesize organic matter and performing transpiration, with the latter providing the driving force for the root system to absorb water and mineral nutrients from the soil [27]. In previous research, humic acid combined with nano-NPK fertilizer promoted leaf area growth in apple trees [28], and phenylalanine, nano-potassium fertilizer, or potassium sulfate boosted chlorophyll content in apple leaves [29], while foliar application of zinc and boron significantly promoted photosynthesis in apple leaves [30]. In this experiment, the application of foliar fertilizers significantly promoted the growth of new shoots, boron-containing foliar fertilizers notably enhanced leaf area expansion. Meanwhile, calcium-based foliar fertilizers improved the SPAD value of leaves while boosting net photosynthetic rate, transpiration rate, stomatal conductance, and intercellular CO_2_ concentration. These results were generally consistent with previous research findings.

Soluble sugars, as one of the leading indicators for evaluating apple fruit quality, is affected by many factors. In this study, the soluble sugar content following amino acid foliar fertilizer treatment increased by 8.53% compared to the control, a result which is largely consistent with previous findings [31]. Additionally, the contents of glucose, sucrose, and sorbitol were also elevated under calcium foliar fertilizer treatment relative to the control [32]. Furthermore, Khan found that compared with the unfertilized control group, the application of amino acid foliar fertilizer at the blooming and fruit-sitting periods had a promoting effect on the content of reducing sugars in Golden tangerine [33], and the total soluble solids, total sugars and total carbohydrates of mango fertilized with amino acid foliar fertilizer were higher than those of the control group by 3.0%, 5.8% and 15.0%. Calcium spraying increased soluble sugar and soluble solids content in mandarin fruits and promoted the accumulation of soluble sugars in the fruits [19]. Cherry variety ‘Sunburst’ produced maximum sugar content after boron spraying [34]. These conclusions are generally consistent with the results of this experiment, even if these fruits have rather different development and maturation features.

Organic acids are important in determining the flavor of fruits, mainly malic, citric, tartaric, oxalic, and quinic acids, to which vitamin C also belongs. After spraying amino acid foliar fertilizers, titratable acid content and vitamin C content of citrus were increased [31]. It was found that application of calcium fertilizer increased vitamin C content and decreased titratable acid content in tomato fruit [35], and others found that application of calcium fertilizers increased vitamin C content in fruits [36,37]; application of boron increased vitamin C and titratable acid content in strawberries, and sweet oranges [38]; and Aye et al. found that foliar spraying of potassium fertilizer reduced the titratable acid content of citrus fruits [39], but others found that potassium fertilizer spray promoted the accumulation of vitamin C and total acid content in strawberries [40], while improving the acidity level of apple fruits [29]. In this experiment, the contents of vitamin C, oxalic acid, and malic acid increased under potassium fertilizer treatment. In the present research, the content of vitamin C under calcium foliar fertilizer treatment was 32.39% higher than that of the control group. Amino acid treatment increased the contents of vitamin C and oxalic acid in fruits, which was generally consistent with the findings of Ali et al. [41].

The natural antioxidant effect of fruits is closely related to phenolics, which help improve plants organs colour and flavor due to their extreme antioxidant activity. The results of this experiment demonstrated that the contents of total phenol, tannin, and anthocyanin increased in ‘Snick’ apples across all treatments. Specifically, the total phenol and tannin contents in the amino acid foliar fertilizer treatment were 25.38% and 47.01% higher than those in the CK, respectively; the anthocyanin content in the potassium fertilizer treatment was 73.09% higher than that in CK; and the flavonoid content in the boron fertilizer treatment increased by 70.25%. This finding is consistent with the results reported by Gao Xiang et al. [4], who observed that spraying mixed amino acids at a concentration of 75 mg·L^−1^ promoted the total phenolic content, total flavonoid content, and total antioxidant activity in celery plants. Other researchers have similar conclusions, such as spraying of boron fertilizer increased the antioxidant properties (total phenols) of strawberry fruits and similarly spraying of potash fertilizer promoted an increase in phenolic compounds and antioxidant activity in strawberry fruits [42].

This study investigated the effects of different nutritional foliar fertilizers on the physiological functions of apple leaves, fruit quality, and the content of aromatic compounds. Among them, mineral source fulvic acid potassium has the most significant improvement on fruit quality. This could be attributed to potassium (T4) involvement in over 60 enzyme system activations, photosynthesis, the transport of assimilated products, carbohydrate metabolism, and protein synthesis [43], thereby promoting significant improvements in fruit quality. But because the absorption capacity and sensitivity of apple trees to different nutrients are different, and the mechanism of related nutrients in different physiological metabolic pathways is different, the effects of amino acid, calcium, boron and potassium foliar fertilizers on the quality of apples are also different.

Principal component analysis and correlation analysis indicate that foliar fertilizers act on crops through leaf absorption and influence fruit quality. The core mechanisms involve direct supplementation of key nutrients, regulation of physiological metabolism, and enhancement of stress resistance [44,45]. Ultimately, these processes optimize both fruit internal components (e.g., sugars, vitamins, and proteins) and external traits (e.g., color, size, and firmness) across three interconnected dimensions: “nutrition supply–physiological activity–environmental adaptation [46].” However, the specific metabolic pathways and regulatory mechanisms remain unknown and have not been thoroughly investigated. In subsequent research, we will place greater emphasis on elucidating the mechanisms of action of foliar fertilizers, as well as evaluating the efficacy of novel nano-formulated foliar fertilizers.

## 4. Materials and Methods

### 4.1. Experimental Site and Experimental Materials

This study was conducted at the Apple Production Dispatch Centre in Ning County, located in the southern part of Qingyang City, Gansu Province. The region experiences a warm temperate continental monsoon climate, characterized by an altitude of 1074 m, an average annual temperature of 8.7 °C, and an average annual precipitation of 565.9 mm. The climate is semi-arid with rainfall concentrated primarily in July, August, and September. Additionally, the area has an average annual evaporation rate of 1442.6 mm, a frost-free period lasting 168 d, and 2369 h of sunshine annually. The terrain of the experimental site is flat, providing favorable conditions for drainage and irrigation. During the experiment, fertilizer application, irrigation water quantity, and irrigation timing were strictly adhered in accordance with the guidelines in the “Fertilizer Application Plan and Drip Irrigation Quota” developed by the Ning County Apple Production Dispatch Centre.

The test material consisted of 6-year-old ‘Snick’ apples planted at a spacing of 1 × 3.5 m, exhibiting a spindle shape and rootstock of M9T337. These trees had identical growth potential and were managed uniformly regarding pruning, fertilization, water management, soil care, and pest and disease control. Composted organic fertilizer (derived from crop residues, cow dung, straw, etc.) was applied as basal fertilizer in autumn at a rate of 45,000 kg·ha^−1^, along with urea (45 kg) and superphosphate (120 kg). An additional topdressing was applied before budburst, consisting of diammonium phosphate (45 kg·ha^−1^) administered via hole application. A second topdressing was applied during the fruit expansion stage, which included potassium chloride (60 kg·ha^−1^) and diammonium phosphate (7.5 kg·ha^−1^), also applied by hole placement.

### 4.2. Experimental Treatment and Design

The experimental treatment is shown in Table 1:

The experiment commenced in June 2022 during the fruit expansion period. The initial spraying occurred at 55 days after full bloom, and was followed by subsequent applications every 15 days, totaling three sprays. The spraying solution was diluted to the appropriate concentration and applied evenly to the foliage from top to bottom until runoff. Applications were conducted at 10:00 a.m. on sunny, windless days to ensure no rainfall within 48 h. A single-factor, completely randomized block design was employed, with 15 healthy and uniformly grown trees assigned to each treatment. Each treatment had three replicates, each consisting of five trees, for a total of 225 labeled trees.

Fruit collection was conducted on 20 August. Fruits of uniform size and without mechanical damage were selected from the four cardinal directions (east, west, south, and north) at the midpoint of the tree crown to minimize sampling errors. For each treatment, 10 fruits were sampled, with three replicates, resulting in a total of 150 fruits. These were immediately stored in a cold storage facility (0 °C) at the Ningxian Apple Production and Distribution Center. Subsequently, the peel and pulp were separated, frozen in liquid nitrogen, transported to the laboratory, and stored in an ultra-low temperature freezer at −80 °C.

### 4.3. Growth Index of New Shoots

Ten new shoot branches of uniform height were selected for each treatment, oriented in four different directions: east, west, south, and north, with three replicates. The length of the branches was kept consistent. The branches were labeled, and a vernier caliper was used to measure the growth length of the new shoots and basal diameter of new shoot. Each measurement was repeated three times per treatment.

### 4.4. Indices of Leaf Growth and Photosynthetic Characteristics

For each treatment, ten leaves were randomly selected with three replicates. The 7th to 9th leaves from the base of the 1-year-old sunny branches were chosen, avoiding the main veins and leaf edges. The SPAD value of the leaves was measured using a hand-held TYS-B portable SPAD chlorophyll content tester (Zhejiang Topyun Agricultural Science and Technology Co., Ltd., Hangzhou, China), with three measurements taken per leaf. Leaf area was measured using a TPYX-A instrument (Zhejiang Tuopu Yunnong Science and Technology Co., Ltd., Hangzhou, China), selecting ten leaves per treatment and repeating the process three times.

Three fruit trees were selected for each treatment, with measurements were made on ten functional leaves still attached to the branch. Photosynthetic parameters, including net photosynthetic rate (Pn), transpiration rate (E), stomatal conductance (gs), and intercellular CO_2_ concentration, were measured using a 3051D portable photosynthetic analyzer (Zhejiang Topyun Agricultural Science and Technology Co., Ltd., Hangzhou, China).

### 4.5. Determination of Fruit Appearance Quality

The weight of individual fruits was measured using an electronic balance, and the weight of each fruit was recorded. An electronic vernier caliper measured the fruit length and transverse diameters, and the fruit shape index was the ratio of the maximum longitudinal diameter to the transverse diameter. The GY-4-J digital hardness tester measured fruit firmness. The L*, a* and b* values were measured by colorimeter (3nh) (Shenzhen 3NH Technology Co., Ltd., Shenzhen, China). The L* value (0–100) represents the brightness of the fruit. The higher the L* value, the whiter and brighter the fruit surface. The a* value (−80–100) represents the red-green hue, with positive values indicating red and negative values indicating green. The larger the absolute value of a*, the darker the fruit surface color. The b* value (−80–70) represents the yellow-blue hue, with positive values indicating yellow and negative values indicating blue.

### 4.6. Determination of Soluble Solids, Soluble Sugar, Titratable Acidity, and Sugar-Acid Ratio

Soluble solids contents were measured using a handheld PAL-1 digital refractometer (Atago Co., Ltd., Tokyo, Japan). The determination of total soluble sugars was performed using the modified anthrone colorimetric method [47]. The titratable acid content was determined using the sodium hydroxide titration method [48]. Then the sugar-acid ratio was calculated as soluble sugar content/titratable acid content.

### 4.7. Determination of Soluble Protein, Vitamin C, pH and Relative Water Content

Fruit soluble protein was measured according to the method described by Wei Jinmei with some modifications [49]. Vitamin C content was analyzed using the spectrophotometric method described [50]. The vitamin C content was determined using a standard curve generated from an ascorbic acid solution. The pH was measured using an acidometer (Lei, PHS-3E) (Shanghai LeiCi, Shanghai, China). The relative water content was determined by heating and drying method. The apple pulp was dried at 105 °C for 12 h and weighed every 2 h. Constant weight was considered achieved when the difference between two consecutive weighing results was ≤0.1 g.

### 4.8. Determination of Total Phenols, Tannins, Anthocyanins and Flavonoids

The total phenol was determined by Folin-Ciocalteu method [51], the tannin content was determined by Folin-Denis method [52], and the anthocyanin and flavonoid were determined by spectrophotometer metric method [53].

### 4.9. High-Performance Liquid Chromatography (HPLC) Analysis of Sugars and Organic Acids

The contents of major organic acids and sugars in fruit pulp were measured according to the method [54], by using high-performance liquid chromatography (HPLC) (Waters Corp., Wilford, MA, USA). Each experiment was carried out with three biological replicates.

### 4.10. Determination of Aroma Substances and Their Thresholds

Aroma substances were collected using headspace solid-phase microextraction (HS-SPME). Specifically, 5.0 g of fruit was weighed into a 50 mL sample vial, followed by the addition of 2.0 g NaCl, 20 μL of internal standard (2-octanol, 8.19 mg·L^−1^), and a magnetic stir bar. The vial was then placed on a magnetic stirrer, where the sample was stirred at 500 r·min^−1^ and heated at 55 °C for 30 min to achieve equilibrium. After this conditioning step, the SPME fiber was inserted into the headspace of the vial, and extraction was carried out for 30 min under continuous stirring and heating at 55 °C. Then were determined by gas chromatography-mass spectrometry [55], VOCs were identified and quantified using an Agilent Model 8860 GC (Agilent Technologies Inc., Santa Clara, CA, USA) and a 5977B mass spectrometer (Agilent) (Agilent Technologies Inc., Santa Clara, CA, USA), both equipped with a 30 m × 0.25 mm × 0.25 μm HP-5MS capillary column. Helium was the carrier gas at a 1.0 mL·min^−1^ linear velocity. The injector temperature was kept at 230 °C, and the detector at 250 °C. The oven temperature was programmed from 40 °C (4 min), increasing at 8 °C·min^−1^ to 180 °C, at 20 °C·min^−1^ to 230 °C, at 5 °C·min^−1^ to 250 °C, and held for 5 min. Mass spectra were recorded in electron impact (EI) ionization mode at 70 eV. Identification of VOCs was achieved by comparing the mass spectra with the data system library (MWGC or NIST) and linear retention index (RI, determined by alkane C5–C40), referencing RI information to increase qualitative accuracy and eliminating the interference of false positive substances.

The following ROAV formula was developed based on the relative concentrations of various aroma components and their respective threshold values in water: ROAV = (C_n_/C_max_) × (T_max_/T_n_) × 100. Here, C_n_ represents the relative content of any VOC (μg·g^−1^), C_max_ represents the component with the highest relative content (μg·g^−1^), T_n_ represents the threshold of any VOC (μg·g^−1^), and T_max_ represents the threshold of the component with the largest relative content (μg·g^−1^) [56].

### 4.11. Data Processing

Data processing and figure generation were performed using Excel 2021 (Microsoft, Redmond, WA, USA). Graphs were created using Origin 2024 (Origin Inc., San Francisco, CA, USA). Statistical analyses, including significance testing and one-way ANOVA, were conducted using SPSS 26.0 (SPSS Inc., Chicago, IL, USA), with a significance level of *p* < 0.05.

## 5. Conclusions

**Figure 9:**
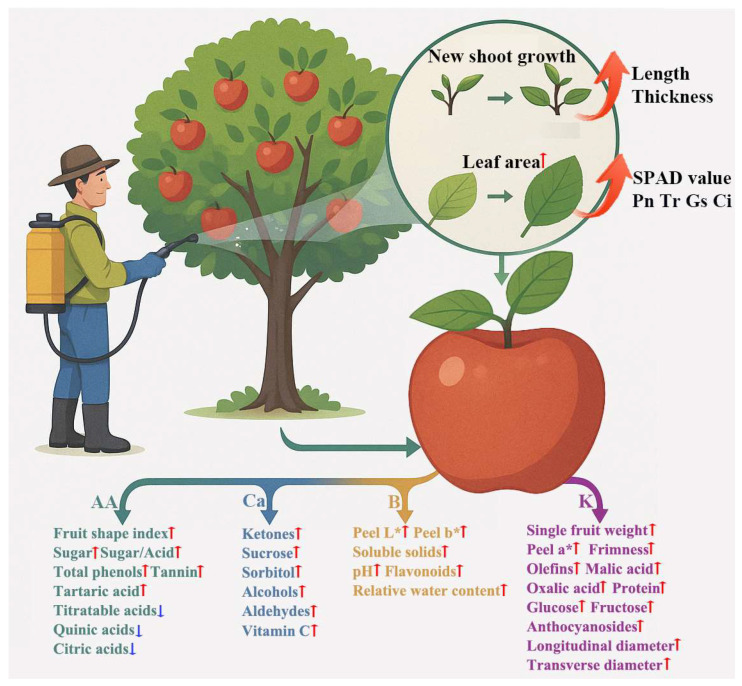
Effects of foliar fertilizer on leaf, fruit quality and aroma content. AA: amino acid foliar fertilizer. Ca: Calcium foliar fertilizer. B: Boron foliar fertilizer. K: Foliar potassium fertilizer. The red upward arrow represents an increase in its content, and the blue downward arrow represents a decrease in its content.

This study demonstrated that foliar fertilization has the potential to improve fruit quality in ‘Snick’ apples (e.g., higher sugar-acid ratio and phenolic content) by enhancing physiological functions such as leaf photosynthetic performance (Figure 9). Although positive effects of foliar fertilizers, particularly mineral source potassium fulvic acid, were observed, it should be noted that fruit load and harvest date were identified as key covariates influencing intrinsic fruit quality and maturity. A higher fruit load may lead to nutrient dilution, which could partially offset the benefits conferred by foliar fertilization. Furthermore, the timing of harvest directly affects fruit acidity, firmness, and metabolite accumulation. These factors may interact with foliar fertilizer treatments and potentially confound the results obtained in this study.

Therefore, the conclusions regarding the efficacy of foliar fertilizers require further validation in subsequent experiments that rigorously control fruit load and incorporate multiple harvest time points. Provided these variables are effectively managed, mineral source potassium fulvic acid can be recommended as a preferred foliar treatment for enhancing ‘Snick’ apple production efficiency. Its application should also be prioritized in further trials involving other apple varieties.

## Figures and Tables

**Table 1:** Basic information of experimental treatments.

Treatment	Foliar Fertilizer	Spray Concentration	Active Principle	Producer	Price (1000 g)
T1	Ye Xiumei	0.2 g·L^−1^	amino acid content of 100 g·L^−1^, Cu + Fe + Mn + Zn + B ≥ 20 g·L^−1^	Qingdao Qianhechun Biotechnology Co., Ltd., Qingdao, China	50 yuan
T2	Fluid calcium	0.067 g·L^−1^	Ca ≥ 180 g·L^−1^, sugar alcohol ≥ 50 g·L^−1^	Shandong Ruipu Biotechnology Co., Ltd., Zouping, China	30 yuan
T3	Sugar alcohol boron	0.05 g·L^−1^	B ≥ 160 g·L^−1^	Shandong Ruipu Biotechnology Co., Ltd., Zouping, China	45 yuan
T4	Mineral source potassium fulvic acid	0.05 g·L^−1^	soluble potassium fulvic acid ≥ 50%, and K_2_O ≥ 12%	Shandong Ruipu Biotechnology Co., Ltd., Zouping, China	75 yuan
CK	water	/	/	/	/

‘/’ means ‘none’ or ‘not applicable’.

## Data Availability

Data are contained within the article.
